# Treating patients with severe mental illness with narrative exposure therapy for comorbid post-traumatic stress disorder

**DOI:** 10.1192/bjo.2020.124

**Published:** 2020-12-09

**Authors:** Maria W. Mauritz, Betsie G.I. van Gaal, Peter J.J. Goossens, Ruud A. Jongedijk, Hester Vermeulen

**Affiliations:** GGNet Center for Mental Health Care, The Netherlands; Radboud University Medical Center, Radboud Institute for Health Sciences, IQ Healthcare, The Netherlands; Radboud University Medical Center, Radboud Institute for Health Sciences, IQ Healthcare, The Netherlands; HAN University for Applied Sciences, Nursing Studies, Nijmegen, The Netherlands; Dimence Group, Center for Mental Health Care, Specialistisch Centrum Bipolaire Stoornissen, The Netherlands; and University Centre for Nursing and Midwifery, Department of Public Health and Primary Care, Faculty of Medicine and Health Sciences, Ghent University, Belgium; ARQ Centrum ’45; and ARQ National Psychotrauma Center, The Netherlands; Radboud University Medical Center, Radboud Institute for Health Sciences, IQ Healthcare, The Netherlands; and HAN University for Applied Sciences, Nursing studies, Nijmegen, The Netherlands

**Keywords:** post-traumatic stress disorder, severe mental illness, narrative exposure therapy, single group, repeated-measures

## Abstract

**Background:**

Interpersonal trauma and post-traumatic stress disorder (PTSD) in patients with severe mental illness (SMI) negatively affect illness course. Narrative exposure therapy (NET) is effective in vulnerable patient groups, but its efficacy and applicability has not been studied in out-patients with SMI.

**Aims:**

We aimed to evaluate the efficacy and applicability of NET in SMI on changes in PTSD, dissociation, SMI symptoms, care needs, quality of life, global functioning and care consumption.

**Method:**

The study had a single-group, pre-test–post-test, repeated-measures design and was registered in The Netherlands National Trial Register (identifier TR571). Primary outcomes were assessed at pre-treatment (T0), 1 month post-treatment (T1) and 7 months’ follow-up (T2), with a structured interview for PTSD and dissociation screening. Secondary outcomes followed routinely SMI measurements and medical data. Mixed models were used for data analysis.

**Results:**

The majority of the 23 participants was female (82%). Mean age was 49.9 years (s.d. 9.8) and mean PTSD duration was 24.1 years (s.d. 14.5). Mean PTSD severity decreased from 37.9 at T0 to 31.9 at T1 (−6.0 difference, 95%CI −10.0 to −2.0), and decreased further to 24.5 at T2 (−13.4 difference, 95%CI −17.4 to −9.4). Dissociation, SMI symptoms, duration of contacts, and medication decreased; global functioning increased; and quality of life and perceived needs did not change. Eleven participants were in remission for PTSD at T2, of which five were also in remission for major depression.

**Conclusions:**

NET appeared efficacious and applicable to out-patients with SMI and PTSD, and was well tolerated.

In the past decade, the high prevalence of interpersonal trauma and post-traumatic stress disorder (PTSD) in patients with severe mental illness (SMI) has received considerable attention. Trauma and PTSD are still underdiagnosed in patients with SMI, and have a negative influence on the illness course,^1–3^ specifically for psychotic disorders,^4^ bipolar disorders^5^ and major depressive disorders.^6^ That is why clinicians and researchers emphasise the importance of adequate screening for trauma and PTSD to counteract undertreatment in this vulnerable population.^7^ Controlled intervention studies have shown that PTSD in patients with SMI can be treated effectively by the following trauma-focused treatment (TFT) options: cognitive–behavioural treatment, prolonged exposure (PE) and eye movement desensitisation reprocessing (EMDR).^8–12^ An alternative TFT is narrative exposure therapy (NET) for patients who are exposed to repeated traumatic events during their life cycle. NET integrates prolonged exposure into the life story and includes attention to positive meaningful events.^13,14^ NET has been proven particularly effective in vulnerable patient groups such as refugees^15,16^ and patients with a history of interpersonal trauma including child and adult abuse.^17^ At this moment, NET has not yet been studied in patients with SMI who are receiving community mental healthcare. Therefore, to underpin the use of NET in clinical practice, this study evaluates the efficacy and applicability of NET in out-patients with SMI with comorbid PTSD associated with repeated interpersonal trauma.

## Method

### Design

This study was a single-group, pre-test–post-test study with repeated-measures, and was part of a larger mixed-methods study.^14^ The study was registered in The Netherlands National Trial Register (identifier TR5714).^18^

### Ethics statement

The authors assert that all procedures contributing to this work comply with the ethical standards of the relevant national and institutional committees on human experimentation and with the Helsinki Declaration of 1975, as revised in 2008. All procedures involving patients were approved by the Committee on Research Involving Human Subjects, Arnhem-Nijmegen (approval number 1843–2015).^14^ The study started in April 2016 and ended in January 2019.

### Participants

We included adult (age 21**–**65 years) out-patients with SMI and a history of repeated interpersonal trauma and comorbid PTSD, who received NET in a community mental healthcare context. The inclusion criteria were out-patients with (a) the existence of SMI, defined as the presence of a bipolar, major depressive, schizophrenia spectrum or personality disorder, according to the Mini-International Neuropsychiatric Interview (MINI-plus)^19,20^ and/or the Structured Clinical Interview for DSM-IV Personality Disorders (SCID-II),^21^ with reduced global functioning according the Global Assessment of Functioning (GAF; score <60)^22,23^ during ≥2 years, according to chart diagnosis; (b) a trauma history including repeated physical and/or sexual abuse according to the Life of Events Checklist for DSM-5 (LEC-5);^24,25^ and (c) the existence of PTSD according to the Clinical-Administered PTSD Scale for DSM-5 (CAPS-5).^26-28^ The exclusion criteria were out-patients with (a) provision of other trauma-focused treatment within 12 months before the study; (b) antisocial personality disorder; (c) dissociative identity disorder or (d) the provision of involuntary treatment following the Dutch Mental Health Law.

### Recruitment

The study was carried out in 10 local flexible assertive community treatment (FACT) teams^29^ located in five geographic regions of a large mental health centre in The Netherlands. FACT team professionals (mainly psychiatrists and specialised psychiatric nurses) screened their patients for trauma and PTSD with the Trauma Screening Questionnaire (TSQ).^30^ After positive screening on the TSQ, the PTSD Checklist for the DSM-5 (PCL-5) was used to verify all PTSD symptoms,^30-32^ and 27 patients with repeated interpersonal trauma and PTSD were selected.

Patients received oral information about NET from their therapists and were asked to participate in the study. The researcher then provided written information and called after 1 week to check whether information was clearly explained and ask for oral consent. Written consent was obtained from all patients before the inclusion interview. Considering the vulnerable position of patients with SMI, FACT team members who knew their patients, were asked to recruit possible eligible patients. They were informed that they could stop with NET and/or participating in the study at any time, without reason. Information included a statement that personal and clinical data were processed anonymously.^33^ There was no reward for participating. The recruitment lasted from April 2016 to January 2018.

### Intervention

The NET was conducted according to the Dutch NET manual,^34,35^ which is based on the manual outlined by Schauer et al.^13^ NET was offered by five therapists (three nurse practitioners and two clinical psychologists) in a maximum of 16 weekly sessions^14^ from May 2016 to October 2018. The therapists were recruited from different FACT teams at the mental health centre and followed a 3-day NET training by qualified trainers in 2015–2016.^36^ They completed additional group video supervision in 10 90-min sessions by a trained NET supervisor during the study. FACT was continued during NET, and comprised the usual coordinated, multidisciplinary treatment interventions for out-patients with SMI, including pharmacotherapy alongside collaborative care, which comprised case management, crisis interventions and outreach nursing care.^37,38^ During NET treatment, minimal biweekly supportive interventions by other FACT team members was requested as an important condition, because of the vulnerability of patients with SMI. In this period, the patients with SMI did not receive any other psychological treatment or benefits.

### Outcomes

The primary outcomes were PTSD and dissociative symptoms. Remission of PTSD was based on the number and severity of symptoms, according to CAPS-5 rules.^26-28^ The secondary outcomes were SMI symptoms, care needs, quality of life, global functioning and care consumption.

### Assessments

At baseline, demographic data were collected via the electronic patient record (EPR) and included gender, age, marital status, cultural background, education, living condition and employment. Clinical characteristics consisted of the verification of the primary SMI diagnosis, duration of illness, suicide risk, suicide attempts and substance misuse were collected with the MINI-plus.^19,20^ During the study, it was decided to monitor long-term changes in diagnosis, substance misuse and suicide risk by a second assessment with the MINI-plus at follow-up (T2). Therefore, additional ethical approval was obtained from the Committee on Research Involving Human Subjects, Arnhem-Nijmegen. The number and duration of interpersonal traumatic event types were collected with the LEC-5.^24,25^ PTSD subtype and duration information were collected according to the CAPS-5.^26,27^

Data for the primary and secondary outcomes were collected at three time points: at baseline (T0), and 1 month (T1) and 7 months (T2) after NET. PTSD symptoms and severity were assessed with the CAPS-5,^26,27^ and dissociative symptoms were assessed with the Dissociative Experiences Scale (DES).^39^ SMI symptoms were assessed with the Health of the Nation Outcome Scale (HoNOS),^40^ care needs were assessed with the Camberwell Assessment of Needs (CAN),^41^ quality of life was assessed with the Manchester Short Assessment of Quality of Life (MANSA)^42^ and global functioning was assessed with the GAF.^23^ These assessments were performed by a trained and independent research assistant, who was supervised by the first author. The HoNOS was assessed by the primary care provider because this instrument is focused on long-term observation. For detailed information on content, validity and reliability of the mentioned diagnostic instruments, consult the study protocol.^14^

Care consumption was defined as the number of therapeutic contacts, including duration in minutes and prescribed medications. These data were collected via the EPR by the first author from 6 months before T0 up to and including T2. The prescribed medications comprised four groups of psychiatric medication. For each group, a standard equivalent was calculated for: (a) benzodiazepines: diazepam;^43^ (b) antidepressants: fluoxetine;^44,45^ (c) antipsychotics: haloperidol^46,47^ and (4) mood stabilisers: topiramate, because this medication was most commonly prescribed.^48^ The cumulative dose for each group of medication was calculated for each participant in the defined periods.

### Sample size and statistical methods

The intended sample size was 25 participants. This size was based on the clinical feasibility of performing the intervention in the mental health centre. It was assumed that each of the five therapists in their geographic region could provide NET to five participants within 2 years. A sample size was not calculated because the great degree of uncertainty, but the chosen number is in line with other feasibility trials with a continuous end-point.^49,50^

To describe demographics and clinical characteristics at baseline, means (s.d.) or median (interquartile range) were used for continuous data, depending on whether there was a normal distribution. Percentages were obtained for categorical data.

Because of the hierarchical structure of our study (repeated-measures nested within participants) we performed a linear mixed models for the analysis of the continuous course of illness outcomes. The duration of contacts had a skewed distribution and therefore a log transformation was performed. The log-transformed outcome was also analysed with a linear mixed model. The number of contacts was analysed with a generalised linear mixed model with a negative binomial distribution. For all these analyses, we used a model with a random intercept and all other variables fixed. A *P*-value of <0.05 was considered to be statistically significant, based on two-sided tests. The analysis was done with IBM SPSS Statistics version 25 for Windows. Table 2 presents back-transformed results.

For the use of psychiatric medication, the cumulative dose was calculated for the following periods: 6 months before T0; between T0 and T1; and 6 months thereafter, at T2. Medications were prescribed once a month to once every 3 months. Therefore, dosages were expressed in daily per month.

## Results

### Participant flow

**Fig. 1:**
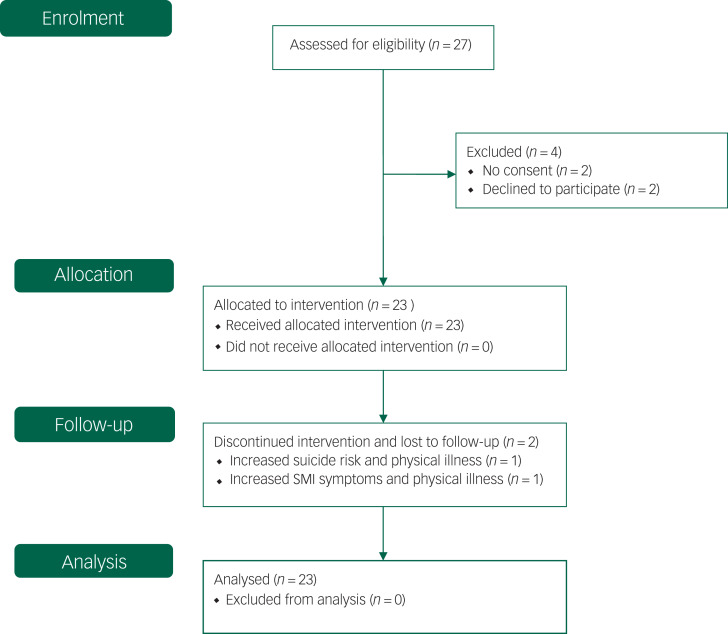
Flow diagram, adapted to single-group design (CONSORT 2010).^51^ SMI, severe mental illness.

In total, 27 eligible patients were contacted and all accepted NET treatment. One man (age 59 years) and one women (age 56 years) received NET, but did not consent to participate in the study. The other 25 patients did consent to participate in the study. Of these, two patients (both female, ages 23 and 39 years) withdrew before inclusion because of somatic illness and family circumstances, respectively. The remaining 23 patients started with NET within 1 week after inclusion. Most of them had never received TFT in the past. Two female patients dropped out during NET, but no serious events occurred (Fig. 1).

### Baseline demographics and clinical characteristics

The mean age of the participants (*n* = 23) was 49.9 years (s.d. 9.81). The majority were women (*n* = 19). Four participants had a non-Western cultural background. Of all of the participants, 14 had a middle education (above elementary school), two had a high education (vocational and academic) and only one was employed. Twelve participants were married or lived together, seven lived alone and four were living in sheltered housing.

Clinical characteristics show that the participants experienced 3–8 years (median) emotional, physical and/or sexual abuse during childhood and/or adulthood. The mean duration of PTSD was 24.1 years (s.d. 14.48).

Major depressive disorder was overrepresented among the participants (*n* = 15), in contrast to schizophrenia spectrum disorder (*n* = 4) and bipolar disorder (*n* = 4). Personality disorder did not appear in this group. The mean duration of SMI was 26.2 (s.d. 12.2). Thirteen participants had attempted suicide in the past and ten participants had current high suicide risk. Substance misuse was relatively low. See Table 1 for further details.

**Table 1:** Demographic and clinical characteristics (*n* = 23)

		*N*	%
Demographics			
Age, years, median and mean (s.d.)		49.9	(9.81)
Gender	Female	19	82.6
Cultural background	Dutch	18	78.3
	Western non-Dutch	1	4.3
	Non-Western	4	17.4
Education	Low	7	30.4
	Middle	14	60.9
	High	2	8.7
Employment	Employed	1	4.3
	Sheltered employed	8	34.8
	Unemployed	14	60.9
Living condition	Married/cohabiting	12	52.2
	Alone	7	30.4
	Sheltered housing	4	17.4
Clinical characteristics			
SMI diagnosis (MINI-plus)	Schizophrenia spectrum disorder	4	17.4
	Bipolar disorder	4	17.4
	Major depressive disorder	15	65.2
	Duration SMI, years, mean (s.d.)	26.2	(12.2)
Current suicide risk (MINI-plus)	No	4	17.4
	Low	6	26.1
	Medium	3	13.0
	High	10	43.5
	Suicide attempt ever	13	56.5
Current substance misuse (MINI-plus)	No	15	65.2
	Drugs	7	30.4
	Alcohol and drugs	1	4.3
Abuse, number, duration, years (LEC-5)		Median	IQR
Childhood (<16 years)	Emotional (*n* = 16)	8.0	5.5
	Physical (*n* = 16)	5.5	8.0
	Sexual (*n* = 12)	3.0	6.0
Adulthood (≥16 years)	Emotional (*n* = 11)	6.0	22.0
	Physical (*n* = 13)	3.0	8.5
	Sexual (*n* = 8)	4.5	8.0
		Mean	s.d.
Reported traumatic events	Total number	8.0	1.52
Post-traumatic Stress Disorder (PSTD) (CAPS-5)	Total number of symptoms	14.0	2.44
	Total severity	40.0	7.70
	Duration, years	24.1	14.48
	Dissociative subtype, *n*, %	5	21.7

MINI-Plus, Mini-International Neuropsychiatric Interview; LEC-5, Life Events Checklist for DSM-5; CAPS-5, Clinician-Administered PTSD Scale for DSM-5.

### Provision of NET

Of the 23 participants who started with NET, two of them discontinued treatment prematurely after one session and four sessions, respectively. The other 21 participants received 8–15 (median 11, interquartile range 2.5) weekly NET sessions. Duration of NET was 10–43 weeks (median 15), including four cases with interrupted treatment because of somatic problems (one participant), family circumstances (one participant), experienced stress from the therapy (one participant) and change of therapist (one participant), but eventually all finished NET after these breaks.

Analysis with mixed models included 23 participants, based on the intention-to-treat principle and taking missing values into account. Twenty-one participants completed the intervention and the study. There was no measurement of one participant at T1, and another at T2. Also missed were two HoNOS measurements at T2. These participants were in remission for PTSD and major depressive disorder and left the mental health centre after treatment; therefore, they could not be observed by the primary care provider. All other measures were completed by the research assistant.

### PTSD and dissociative symptoms

The number of PTSD symptoms and PTSD severity (CAPS-5) decreased over time between baseline, post-treatment and follow-up. The number and severity of each PTSD cluster symptoms decreased over time between baseline and follow-up. Intrusion, avoidance, cognition and mood symptoms decreased during and after treatment, whereas arousal and reactivity were decreased at follow-up (see Table 2). Severity of dissociative symptoms (DES) also decreased between baseline, post-treatment and follow-up. Eight participants were in remission for PTSD at post-treatment and three other participants were in remission at follow-up. Remission rates in the diagnostic subgroups were almost similar: 8 out of 15 for depressive disorder, 2 out of 4 for bipolar disorder and 1out of 4 for schizophrenia spectrum disorder.

**Table 2:** Results of primary and secondary outcomes based on a mixed model analysis for repeated measures of the full analysis set^1^

Outcome	Baseline (T0)	1 month after NET (T1)			7 months after NET (T2)			Overall significance
	Mean (95%CI)	Mean (95%CI)	LSMD (95%CI)	*P-*value	Mean (95% CI),	LSMD (95%CI)	*P-*value	*P-*value
Primary outcomes for PTSS								
CAPS-5, total symptoms (0–20)	13.65 (11.83–15.47)	11.15 (9.25–13.04)	−2.50 (−4.19 to −0.82)	0.005	8.82 (6.93–10.71)	−4.83 (−6.52 to −3.14)	<0.001	<0.001
CAPS-5, total severity (0–80)	37.91 (32.93–42.90)	31.90 (26.76–37.04)	−6.01 (−10.02 to −2.01)	0.004	24.54 (19.40–29.68)	−13.37 (−17.38 to −9.37)	<0.001	<0.001
CAPS-5, total severity clusters								
B, intrusions (0–20)	10.78 (8.95–12.61)	9.01 (7.12–10.90)	−1.77 (−3.33 to −0.22)	0.026	6.56 (4.66–8.45)	−4.23 (−5.78 to −2.67)	<0.001	<0.001
C, avoidance (0–8)	4.00 (3.35–4.65)	2.51 (1.82–3.21)	−1.49 (−2.38 to −0.59)	0.002	1.87 (1.17–2.56)	−2.13 (−3.03 to −1.24)	<0.001	<0.001
D, cognition and mood (0–28)	13.70 (11.66–15.73)	11.16 (9.04–13.29)	−2.53 (−4.52 to −0.55)	0.014	9.69 (7.57–15.73)	−4.00 (−5.99 to −2.02)	<0.001	<0.001
E, arousal and reactivity (0–24)	9.43 (7.79–11.08)	9.01 (7.38–10.82)	−0.34 (−1.90 to 1.23)	0.664	6.38 (4.67–8.10)	−3.05 (−4.61 to −1.49)	<0.001	<0.001
DES, total severity (0–100)	27.79 (21.17–34.42)	22.41 (15.65–29.18)	−5.38 (−9.77 to −0.99)	0.018	20.97 (14.20–27.73)	−6.83 (−11.21 to −2.44)	0.003	0.008
Secondary outcomes for SMI								
HoNOS, total severity (0–48)	12.30 (9.79–14.81)	10.04 (7.34–12.73)	−2.27 (−4.70 to 0.16)	0.067	9.94 (7.31–12.54)	−2.38 (−4.72 to −0.03)	0.047	0.083
CAN, total severity (0–44)	10.22 (8.21–12.22)	10.79 (8.72–12.85)	0.57 (−1.05 to 2.19)	0.482	9.91 (7.84–11.98)	−0.30 (−1.92 to 1.31)	0.705	0.562
MANSA, total quality (12–84)	52.39 (47.77–57.01)	55.38 (50.67–60.09)	2.99 (0.28–5.95)	0.048	53.04 (48.33–57.75)	0.65 (−2.31 to 3.61)	0.660	0.117
GAF, total functioning (0–100)	49.39 (47.00–51.78)	49.24 (46.72–51.77)	−0.15 (−2.93 to 2.64)	0.915	53.39 (50.86–55.91)	4.00 (1.21–6.78)	0.006	0.008
Care consumption^a^								
Contacts per period (number)	35.0 (22.8–53.8)	40.0 (26.0–61.4)	5.0 (−10.8 to 31.1)	0.598	30.6 (19.9–47.0)	−4.4 (−14.6 to 15.7)	0.597	0.572
Duration of contacts (minutes)	2021 (1475–2770)	3749 (3111–4518)	1728 (1163–2388)	<0.001	1271 (803–2010)	−750 (−940 to −526)	0.006	<0.001

NET, Narrative Exposure Therapy; LSMD, least squares mean difference; CAPS-5, Clinician Administered PTSD Scale for DSM-5; DES, Dissociative Experiences Scale; SMI, severe mental illness; HoNOS, Health of the Nation Outcome Scale; CAN, Camberwell Assessment of Needs; MANSA, Manchester Short Assessment of Quality of Life; GAF, Global Assessment of Functioning scale.

a. Results for the number and duration of contacts (obtained from the electronic record) are based on analyses on a logarithmic scale; table presents back-transformed results. The columns T0, T1 and T2 refer to the period before, during and after treatment.

### SMI symptoms, care needs, quality of life and global functioning

Severity of SMI symptoms (HoNOS) decreased between baseline (T0) and follow-up (T2). No significant changes occurred in quality of life (MANSA) and care needs (CAN). Global functioning (GAF) increased over time, between post-treatment and follow-up (see Table 2).

Between T0 and T2, suicide risk shifted from high (44% to 35%) and medium risk (13% to 5%) to low risk (26% to 35%). In the group as a whole, substance misuse decreased from 30% to 24%. One participant had alcohol and substance use at baseline, but was abstinent for both at follow-up. Of the 11 participants in remission for PTSD, five were also in remission for major depression at follow-up (MINI-plus).

### Care consumption

The number of contacts changed from 35 in the 6 months before treatment (T0), to 40 during treatment (T0 to T1), and to 31 during the 6 months after treatment (T1 to T2), although this was not statistically significant (overall *P* = 0.572). However, the geometric mean of the duration of the contacts increased 1.85 times (95%CI 1.35–2.55) from 2021 min (34 h) in the 6 months before treatment (T0) to 3749 min (62 h) for the treatment period T1–T0, whereas it decreased by a factor 0.63 (95%CI 0.48–0.86) to 1271 min (21 h) in the 6 months after treatment (T2) compared with the 6 months before treatment (T0; overall *P* = <0.001).

**Fig. 2:**
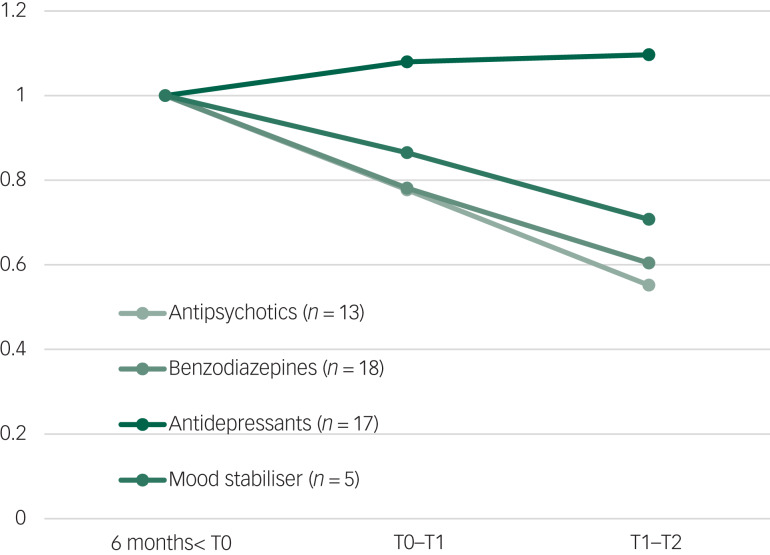
Average medication dose before baseline (6 months before T0), during treatment (T0 to T1) and after treatment (T1 to T2).

Before and during the study, 21 participants received psychiatric medication. Prescribed doses of antipsychotics (*n* = 13) and benzodiazepines (*n* = 16) were the most reduced. Mood stabilisers (*n* = 5) also decreased, but the number of consumers was small. Antidepressant (*n* = 15) doses increased during NET and did not decrease between post-treatment and follow-up. The trends in medication are shown in Fig. 2.

### Harms

No serious events occurred during NET and follow-up. In addition, none of the participants were admitted to hospital or needed crisis management during the study.^14^

## Discussion

### Main findings

To the best of our knowledge, this is the first study to examine the feasibility and applicability of NET for comorbid PTSD in out-patients with SMI. PTSD symptoms and severity reduced significantly at post-treatment and follow-up. All four PTSD clusters showed significant reduction of symptoms, whereas intrusions, arousal and reactivity decreased more slowly than symptoms of avoidance, cognition and mood. These results are in line with other controlled NET studies in vulnerable patients, such as children and adolescents,^52^ patients with borderline personality,^17^ the elderly,^53^ refugees^15^ and asylum-seeking refugees with PTSD and depressive disorder.^54^ Of the 23 participants, 11 (48%) were in remission for PTSD at follow-up. This corresponds to other NET studies that reported 38%, 55% and 71%.^55,56^

Our results suggests that patients with SMI may benefit from NET just like other patient groups. So far, NET has not studied specifically in patients with SMI. Until now, most NET studies excluded patients with psychosis, bipolar disorder, substance misuse and suicidal ideations.^17,52-54^ One case study reported positive results of NET in a refugee with PTSD and psychotic features.^57^ Mørkved *et al*^55^ compared NET and prolonged exposure therapy for PTSD and argued that NET and prolonged exposure have several commonalities when it comes to the principles of exposure. An important feature of NET is that trauma-processing is never an isolated event, but is always embedded in the context of a traumatic event and in the life history as a whole. Given this focus on the autobiographical elaboration of traumatic experiences, NET is particularly suited for populations with multiple trauma and complex mental health conditions.^16^

Comparing NET with prolonged exposure makes sense to the extent that it has shown to be effective in patients with SMI and psychotic disorders, including schizophrenia, bipolar and depressive disorders, in a controlled study. Dissociation, substance use and suicidality risk were not excluded in this study. Results showed that there was no increase of hallucinations, dissociation or suicidality.^9,12^ The findings in our study are in line with these results: dissociation, suicidality risk and substance misuse were also not excluded, and all showed reduction during and after NET. Severity of dissociative symptoms also declined significantly at post-treatment and follow-up. These findings are in line with some other NET studies and other TFT studies.^17,56,58^ Also, a meta-analysis on the effect of dissociation suggests that pre-treatment dissociation does not determine trauma-focused psychotherapy outcomes in PTSD.^58^

SMI symptoms were assessed with generic measures to allow for comparisons across diagnostic groups.^59^ The coherence of the SMI outcomes was low: a slight improvement in SMI was seen based on HoNOS, there were no significant changes in perceived care needs (CAN) and quality of life (MANSA) based on self-reporting, and care providers concluded that global functioning (GAF) was significantly increased.

The MINI-plus is a diagnostic and more specific instrument to measure mental disorders, which, in this study, showed remission in 5 out of the 15 participants with major depression, and over all lower disease burden, suicide risk and less substance use. This is partly comparable with the results of a systematic review of outcomes for psychological interventions for PTSD in psychosis. For prolonged exposure and EMDR, secondary outcomes for psychopathology and distress showed significant reduction, especially for depression and anxiety, but social functioning did not improve. The long duration of SMI and additional social isolation are supposed to be influencing factors.^60^

The duration of contacts increased during NET (T0 to T1), but decreased significantly during the 6 months’ follow-up (T1 to T2), which might reflect less burden of disease. Doses of prescribed benzodiazepines, antipsychotics and mood stabilisers also decreased during and after NET. This suggests that patients experienced less anxiety, irritability, arousal, reactivity and showed reduction of psychotic and bipolar symptoms. In contrast, doses of antidepressants slightly increased during NET and did not change at follow-up. Antidepressants were indicated for both depression and PTSD, and are more difficult to phase out, particularly selective serotonin reuptake inhibitors.^61^ To our knowledge, this is the first study on psychological interventions for PTSD in SMI that takes into account care consumption, expressed as contacts and prescribed medication.^60,62^

### Findings in context: treating PTSD in out-patients with SMI

Our results support the notion that out-patients with SMI with comorbid PTSD can tolerate intensive trauma-focused therapy like NET. The long-standing clinical perception of vulnerability in patients with SMI has not only resulted in appropriate psychiatric care, but unfortunately in the underestimation of the resilience of these patients. This perception may influence underdiagnosis, overlooking trauma histories and not recognising comorbid PTSD, all contributing to undertreatment.^1,2,7,63^ Patients and care providers have to balance resilience and vulnerability in careful conversations, to consider the right time and circumstances for treatment. This study was therefore embedded in clinical practice where optimal care and support by FACT team members was an important condition. Given that the overall disease burden was high and TFT is often perceived as intensive, most of the participants could endure and tolerate NET. As no serious adverse events occurred, no crisis management was required and no patients were admitted to hospital, this underlines the applicability and safety of NET in this population.

### Strengths and limitations

A strength of this study is that it was conducted in the real-life clinical context. NET was provided to out-patients with SMI in a familiar environment, with support from their known care providers from the certified FACT teams. Diagnostic assessments (i.e. PTSD and SMI) were in line with clinical practice and, in addition, routine outcome measurements for SMI were used. NET also fits well in the workflow of the involved professional, which is helpful for future implementation. Furthermore, this strategy avoids extra diagnostic burden and ensures its applicability and implementation in clinical practice.

A second strength of the study was that the five trained and certified NET therapists were all FACT team members and experienced with out-patients with SMI. They followed the NET protocol and received group supervision from a trained NET supervisor.^36^ Because of this, they were able to discuss questions and dilemmas among NET for out-patients with SMI.

The use of the EPR to monitor prescribed medication and contacts is a third strength, because these objective data were not influenced by oral assessments.

Despite these strengths, the limitations of this study are that it was conducted in a small study population without a control group. Moreover, most participants were female; therefore caution is warranted for statements about effectiveness for men. However, for all participants together, the observed changes are in line with the proven effect of prolonged exposure, which shares important principles with NET.

Second, generalisability is limited, as no subgroup analysis could be done by age, gender or primary diagnosis. However, prolonged exposure and EMDR have be shown to be effective in psychosis^60^ and SMI,^62^ whereas NET is effective in major depressive disorder.^54^

Third, the used routine outcome measurements are possibly not responsive and distinctive enough with regard to SMI symptoms, care needs and quality of life.^64^ Finally, implementing this intervention in daily clinical practice also meant that the NET protocol and the agreed biweekly FACT support were not always strictly followed. This concerns mostly interruptions during NET and replacement of FACT care providers.

### Interpretation and recommendations

In conclusion, our results support that NET is feasible and applicable to SMI out-patients in a FACT setting. NET seems to be a valuable addition to other evidence-based, trauma-focused therapies (i.e. cognitive–behavioural treatment, prolonged exposure and eye movement desensitisation reprocessing) and is specifically indicated for PTSD related to repeated interpersonal trauma. Given the high prevalence of repeated interpersonal trauma and PTSD in patients with SMI^2,3^ and the burden of disease, offering TFT to these patients is important. Relevant screening by means of structured diagnostic interviews for trauma history, PTSD and primary SMI disorder are recommended in this group. Using more specific diagnostic instruments to evaluate changes in SMI could help to implement appropriate care. These strategies require sufficient training for therapists and supporting FACT team members. In this context, it is helpful and encouraging that national health institutes are increasingly convinced of the importance of developing trauma-informed care policies in mental health systems.^7^

## Data Availability

The data that support the findings of this study are available from the corresponding author, M.W.M., upon reasonable request.

## Supplementary material

For supplementary material accompanying this paper visit http://dx.doi.org/10.1192/bjo.2020.124. click here to view supplementary material
